# Postoperative Serum Creatinine Serves as a Prognostic Predictor of Cardiac Surgery Patients

**DOI:** 10.3389/fcvm.2022.740425

**Published:** 2022-02-16

**Authors:** Jian Hou, Liqun Shang, Suiqing Huang, Yuanhan Ao, Jianping Yao, Zhongkai Wu

**Affiliations:** ^1^Department of Cardiac Surgery, The First Affiliated Hospital of Sun Yat-sen University, Guangzhou, China; ^2^National Health Council (NHC) Key Laboratory of Assisted Circulation, Sun Yat-sen University, Guangzhou, China

**Keywords:** postoperative creatinine, prognosis of cardiac surgery, Medical Information Mart for Intensive Care III (MIMIC-III), glomerular filtration rate (GFR), acute kidney injury (AKI)

## Abstract

**Background:**

Serum creatinine, an important diagnostic indicator for acute kidney injury (AKI), was considered to be a risk factor for cardiovascular disease. This study aimed to investigate the significance of postoperative serum creatinine in predicting the prognosis of cardiac surgery patients.

**Methods:**

The Medical Information Mart for Intensive Care III (MIMIC-III) database was used to extract the clinical data. Adult (≥18 years) cardiac surgery patients in the database were enrolled. The correlation of postoperative serum creatinine with lengths of intensive care unit (ICU) stay was analyzed with Spearman correlation, and the association of postoperative serum creatinine with hospital mortality was analyzed with chi-square tests. Multivariable logistic regression was used to identify postoperative serum creatinine as an independent prognostic factor for hospital mortality.

**Results:**

A total of 6,001 patients were enrolled in our study, among whom, 108 patients (1.8%) died in the hospital. Non-survivors had much higher postoperative serum creatinine levels (initial: 0.8 vs. 1.2 mg/dl, *P* < 0.001; maximum: 1.1 vs. 2.8 mg/dl, *P* < 0.001; minimum: 0.8 vs.1.1 mg/dl, *P* < 0.001). Positive correlations were observed between postoperative serum creatinine (*P* < 0.001) and lengths of ICU stay. For all models, postoperative initial creatinine, postoperative maximum creatinine, and postoperative minimum creatinine were all positively associated with hospital mortality (all *P* < 0.001). The predictive performance of postoperative serum creatinine was moderately good (area under the curve (AUC) for initial creatinine = 0.7583; AUC for maximum creatinine = 0.8413; AUC for minimum creatinine = 0.7063).

**Conclusions:**

This study demonstrated the potential to use postcardiac surgery serum creatinine as an outcome indicator.

## Introduction

Creatinine is the most common indicator of kidney injury. Notably, creatinine is poorly correlated with the results of a variety of clinical conditions, such as end-stage renal disease (ESRD), progressive chronic kidney disease, multiple organ dysfunction syndrome (MODS), uremia, and death (1). As is known to all, the serum creatinine concentration was not only affected by the glomerular filtration rate (GFR), but also by muscle mass, diet, and drugs (2–4). This may cause the creatinine clearance to overestimate the true GFR (2). So it is warned not to use serum creatinine as a diagnostic and prognostic indicator of multifaceted acute kidney injury (AKI). However, creatinine remains a standard for predicting AKI (5). Serum creatinine is an important diagnostic indicator for AKI (6, 7). What is more, continuous measurement confirmed that even a small increase of serum creatinine was correlated with increased mortality (2–4). In general, the serum creatinine level usually increases by 0.1–0.2 mg/dl (8.8–17.7 μmol/l) after cardiac surgery. If the creatinine level increases by ≥0.3 mg/dl (26.5 μmol/l) within 2 days after surgery, the patient is evaluated to have stage 1 AKI according to the Kidney Disease Improving Global Outcomes (KDIGO) criteria (8). Even a slight increase in creatinine has been shown to alter the patient's prognosis, which is often associated with serious consequences (9–11).

The specific relationship between different postoperative serum creatinine levels and patient prognosis is not fully understood. Given that AKI is one of the most common major complications after cardiac surgery, it can cause increased morbidity and mortality (12), so we designed this study to investigate the correlation between different postoperative creatinine (maximum, minimum, initial) and the outcome of cardiac surgery patients.

## Patients and Methods

### Data Source

Data were analyzed retrospectively from a freely accessible critical care database named Medical Information Mart for Intensive Care III (MIMIC-III, version: MIMIC-III v1.4) (13). The database consists of clinical data of patients who stayed in the ICU of Beth Israel Deaconess Medical Center between 2001 and 2012. After online training under the Collaborative Institutional Training Initiative (CITI) program of the National Institutes of Health (NIH), the right to access the database and acquire the data was approved by the Institutional Review Boards of the Massachusetts Institute of Technology (Cambridge, MA, USA) (Record ID 41268972).

### Patient Selection

From all patients in the MIMIC-3 database, patients were included as follows: (1) those who underwent on-pump cardiac surgery; (2) above the age of 18 years; and (3) those with full records of routine preoperative blood examinations within 24 h after surgery, including creatinine, urea, white blood cells (WBCs), platelets, glucose, potassium, and sodium. The exclusion criteria of this study: (1) patients without undergoing on-pump cardiac surgery; (2) blood test indicators were incomplete; (3) the number of serum creatinine tests within 24 h after surgery was <3.

### Data Extraction

All clinical data were queried and extracted by using the Structured Query Language (SQL), and pgAdmin4 was used as the administrative platform for PostgreSQL. The extracted data included: (1) demographics: age and sex; (2) vital signs: systolic blood pressure (SBP), diastolic blood pressure (DBP), heart rate (HR), temperature and percutaneous oxygen saturation (SpO2), respiratory rate (RR); (3) comorbidities: valvular disease, cardiac arrhythmias, congestive heart failure, peripheral vascular disorder, pulmonary circulation disorder, hypertension, chronic pulmonary disease, complicated diabetes, uncomplicated diabetes, renal failure, and liver disease; (4) laboratory events: peripheral white blood cell count, platelet count, serum glucose, serum sodium, serum potassium, and serum creatinine; (5) SOFA scores and SAPS II; and (6) surgical type: coronary bypass artery grafting (CABG), valvular surgery, repair of septal defect of heart, aortic aneurysm surgery, and others. Hospital mortality and lengths of ICU stay were recorded to analyze the outcome. Considering the ratio of <1.5% missing data for each variable, we directly omitted them in further analysis.

The surgical type was identified using International Classification of Diseases, Ninth Revision, Clinical Modification (ICD-9-CM) codes. For GABG, the ICD-90-CM codes were 3610 and 3619. For valvular surgery, the ICD-90-CM codes were 3599, 3500, 3501, 3502, 3503, and 3504. For repair of septal defect of heart, the ICD-90-CM codes were 3550, 3560, 3570, and 3598. For aortic aneurysm surgery, the ICD-90-CM code was 3732. For others, the ICD-90-CM codes were 3710, 3539, 3542, 3733, and 3737.

For serum creatinine, initial creatinine represented the initial value of creatinine detected after surgery. The maximum creatinine represented the maximum value of creatinine detected during the postoperative lengths of ICU stay, and minimum creatinine represented the minimum value of creatinine detected during the postoperative lengths of ICU stay. The creatinine measurement was used the chemical method according to the protocol of Beth Israel Deaconess Medical Center. The detection interval was a random time within 24 h according to the choice of the clinician, and there was no fixed interval. Only patients with creatinine detections ≥ 3 within 24 h after surgery were used for analysis. For other blood indicators, such as urea, WBC, platelets, glucose, potassium, sodium, the average value of all measured values within 24 h after surgery was used.

### Statistical Analysis

Continuous variables are presented as the median (interquartile range) or mean ± SD and were compared by Mann-Whitney *U*-test or *t*-test. Categorical data are presented as numbers with proportions and were analyzed by the χ2 test. The correlations between lengths of ICU stay and the laboratory results were analyzed by using the non-parametric Spearman's rank correlation test. Logistic regression was applied for the univariable and multivariable analyses to identify independent prognostic factors of hospital mortality after cardiac surgery. Three different models were used: Model 1 was adjusted for age, height, Systolic Blood Pressure (SBP), Diastolic Blood Pressure (DBP), Respiratory Rate (RR), Saturation of Peripheral Oxygen (SpO2), temperature, congestive heart failure, cardiac arrhythmias, renal failure, serum urea, hypertension, and liver disease. Model 2 was adjusted for height, age, SBP, DBP, RR, SpO2, temperature, serum urea, cardiac arrhythmias, congestive heart failure, renal failure, hypertension, Sepsis-related Organ Failure Assessment (SOFA) score, valvular disease, and liver disease. Model 3 was adjusted for age, height, renal failure, hypertension, and SOFA score. *P*-values < 0.05 were considered indicative of statistical significance. Receiver operating characteristic (ROC) curves and the area under the curve (AUC) were used to analyze the sensitivity and specificity. All statistical analyses were performed using the STATA, version 14.0 (StataCorp, College Station, TX, USA).

## Results

### Baseline Characteristics of the Study Population

A total of 6,001 patients were included in our study, of which 108 patients (1.8%) died in the hospital. The baseline characteristics of the study population are briefly summarized in Table 1, including demographics, vital signs, scores, comorbidities, and laboratory events.

**Table 1:** Baseline characteristics of the study population with different survival status.

	**Survivors**	**Nonsurvivors**	***P-*** **value**
	**(***n*** = 5,893)**	**(***n*** = 108)**
**Demographics**			
Age	66.62 ± 12.24	71.00 ± 13.39	<0.001
Male, *n* (%)	4088 (69.4%)	64 (59.3%)	0.024
Weight (kg)	81.8 (70.3-94.6)	77.3 (64-93.1)	0.652
Height (cm)	172.72 (162.56-177.8)	167.01 (157.48-177.8)	<0.001
**Vital signs**			
HR, beats/min	84.06 (78.43-90.37)	86.61 (77.16-96.28)	0.0131
SBP, mmHg	111.66 (106.04-118.39)	108.49 (101.96-114.23)	<0.001
DBP, mmHg	56.72 (52.70-61.08)	55.04 (47.84-61.27)	0.009
RR, times/min	16.85 (15.25-18.79)	18.05 (15.40-21.18)	<0.001
Temperature, °C	36.84 (36.53-37.17)	36.59 (36.33-37.04)	<0.001
SpO2, %	98.15 (97.21-98.96)	97.64 (95.59-98.54)	<0.001
**Laboratory events**			
WBC, 10^9^/L	12.1 (9.3-15.5)	13.2 (9.25-16.8)	0.270
Platelets, 10^9^/L	154 (122-196)	170 (109-228)	0.063
Glucose, mg/dl	135 (114-162)	141 (113.5-178.5)	0.002
Serum sodium, mmol/L	137 (135-138)	137 (135-140)	0.221
Serum potassium, mmol/L	4.4 (4-5.1)	4.3 (3.85-5.1)	0.523
Postoperative serum initial creatinine, mg/dl	0.8 (0.7-1)	1.2 (0.9-1.6)	<0.001
Postoperative serum maximum creatinine, mg/dl	1.1 (0.9-1.4)	2.8 (1.7-4.5)	<0.001
Postoperative serum minimum creatinine, mg/dl	0.8 (0.6-0.9)	1.1 (0.8-1.45)	<0.001
Serum creatinine before surgery, mg/dl	1 (0.8-1.2)	1.2 (0.9-1.6)	<0.001
Serum urea, mg/dl	15 (12-20)	22 (17-30.5)	<0.001
**Comorbidities**			
Congestive heart failure	1,486 (25.2%)	54 (50%)	<0.001
Cardiac arrhythmias	2,940 (49.9%)	73 (67.6%)	<0.001
Valvular disease	1,285 (21.8%)	35 (32.4%)	0.008
Pulmonary circulation disorder	431 (7.3%)	13 (12.0%)	0.063
Hypertension	4,164 (70.7%)	54 (50.0%)	<0.001
Chronic pulmonary	1,112 (18.9%)	26 (24.1%)	0.172
Uncomplicated diabetes	1,541 (26.1%)	21 (19.4%)	0.116
Complicated diabetes	330 (5.6%)	10 (9.3%)	0.103
Liver disease	142 (2.4%)	26 (24.1%)	<0.001
Renal failure	465 (7.9%)	20 (18.5%)	<0.001
**Scores**			
SAPS II	33 (27-41)	45 (36.5-56)	<0.001
SOFA	5 (3-6)	8 (4.5-11)	<0.001
**Surgical type**			
CABG	4138 (70.2%)	65 (60.2%)	0.024
Valvular surgery	3 (0.1%)	0 (0%)	0.815
Repair of septal defect of heart	5 (0.1%)	1 (0.9%)	0.006
Aortic aneurysm surgery	15 (0.3%)	2(1.9%)	0.002
Others	264 (4.5%)	7 (6.5%)	0.321

*Values are presented as the mean ± standard deviation, median (interquartile range), or number of patients (%)*.

*HR, heart rate; SBP, systolic blood pressure; DBP, diastolic blood pressure; RR, respiratory rate; SpO2, percutaneous oxygen saturation; WBC, white blood cell; SAPS II, Simplified Acute Physiology Score II; SOFA, Sequential Organ Failure Assessment; CABG, coronary artery bypass grafting*.

As shown in Table 1, non-survivors had a higher average age than survivors (66.62 ± 12.24 vs. 71.00 ± 13.39, *P* < 0.001). More survivors were male, while the difference between the groups was small (69.4 vs. 59.3%, *P* = 0.024). Non-survivors had much higher postoperative serum creatinine levels (initial:.8 vs. 1.2 mg/dl, *P* < 0.001; maximum: 1.1 vs. 2.8 mg/dl, *P* < 0.001; minimum:.8 vs.1.1 mg/dl, *P* < 0.001) (Table 1). Nonsurvivors tended to have lower SBP, DBP, temperature, and SpO2, as well as higher HR, RR, glucose, serum creatinine before surgery, SOFA score, and simplified acute physiology scores II (SAPS II) score, and a history of congestive heart failure, valvular heart disease, hypertension, cardiac arrhythmias, liver disease, and renal failure (Table 1).

### Clinical Outcomes of the Study Population

The correlations of lengths of ICU stay with laboratory events were calculated with the non-parametric Spearman's rank correlation test. The results showed positive correlations between postoperative serum creatinine (*P* < 0.001), serum creatinine before surgery (*P* < 0.001), serum urea (*P* < 0.001), platelets (*P* = 0.007), and glucose (*P* = 0.010) and lengths of ICU stay (Table 2). Furthermore, the relationship of creatinine with hospital mortality was analyzed. In the fourth quartile of postoperative creatinine, hospital mortality significantly increased compared with the other quartiles (Table 3).

**Table 2:** The correlation of laboratory events with lengths of intensive care unit (ICU) stay.

	**Rho**	***P*** **-value**
Postoperative serum initial creatinine, mg/dl	0.165	<0.001
Postoperative serum maximum creatinine, mg/dl	0.320	<0.001
Postoperative serum minimum creatinine, mg/dl	0.052	<0.001
Serum creatinine before surgery, mg/dl	0.140	<0.001
WBC, 10^9^/L	0.005	0.680
Platelets, 10^9^/L	0.035	0.007
Glucose, mg/dl	0.033	0.010
Serum sodium, mmol/L	−0.002	0.865
Serum potassium, mmol/L	−0.042	0.001
Serum urea, mg/dl	0.182	<0.001

**Table 3:** The relationship between postoperative serum creatinine with hospital mortality.

	**Q1**	**Q2**	**Q3**	**Q4**	***P*** **-value**
**Survivors**	2,227 (99.33%)	987 (99.2%)	1,432 (98.96%)	1,247 (94.68%)	<0.001
**Nonsurvivors**	15 (0.67%)	8 (0.8%)	15 (1.04%)	70 (5.32%)	

*Q, Quartile of postoperative serum initial creatinine value*.

A univariable logistic regression was used for analysis. As shown in Table 3, postoperative serum creatinine, serum creatinine before surgery, age, height, DBP, SBP, RR, temperature, SpO2, serum urea, valvular heart disease, congestive heart failure, hypertension, cardiac arrhythmias, renal failure, liver disease, SAPS II score, and SOFA score were associated with hospital mortality (Table 4).

**Table 4:** Univariable logistic regression analyses for hospital mortality in cardiac surgery patients.

**Variable**	**OR (95%CI)**	***P***
Postoperative serum initial creatinine, mg/dl	1.390 (1.257-1.547)	<0.001
Postoperative serum maximum creatinine, mg/dl	1.543(1.434-1.660)	<0.001
Postoperative serum minimum creatinine, mg/dl	1.620 (1.360-1.930)	<0.001
Serum minimum creatinine before surgery, mg/dl	1.323 (1.194-1.464)	<0.001
Age	1.033(1.015-1.051)	<0.001
Height (cm)	0.963 (0.946-0.980)	<0.001
SBP, mmHg	0.953 (0.933-0.974)	<0.001
DBP, mmHg	0.963 (0.936-0.990)	0.009
RR, times/min	1.115 (1.056-1.176)	<0.001
Temperature, °C	0.464 (0.318-0.676)	<0.001
SpO2, %	0.753 (0.689-0.823)	<0.001
Serum urea, mg/dl	1.039 (1.029-1.050)	<0.001
Congestive heart failure	2.970 (2.025-4.344)	<0.001
Cardiac arrhythmias	2.095 (1.396-3.144)	<0.001
Valvular disease	1.719 (1.144-2.585)	0.009
Hypertension	0.415 (0.284-0.608)	<0.001
Liver disease	12.841 (8.014-20.577)	<0.001
Renal failure	2.653 (1.618-4.351)	<0.001
SAPS II	1.063 (1.050-1.076)	<0.001
SOFA	1.377 (1.295-1.465)	<0.001

The multivariable analysis results are shown in Table 4. In the multivariable analysis, Model 1 was adjusted for height, SBP, age, DBP, RR, SpO2, temperature, cardiac arrhythmias, congestive heart failure, renal failure, serum urea, hypertension, and liver disease. Model 2 was adjusted for age, height, SBP, DBP, RR, SpO2, temperature, congestive heart failure, renal failure, cardiac arrhythmias, serum urea, hypertension, SOFA score, valvular disease, and liver disease. Model 3 was adjusted for age, height, renal failure, hypertension, and SOFA score. For all Models, postoperative serum initial creatinine [Model 1: odds ratio (OR) = 1.331, 95%CI = 1.112-1.593, *P* = 0.002; Model 2: OR = 1.339, 95%CI = 1.116-1.607, *P* = 0.002; Model 3: OR = 1.361, 95%CI = 1.198-1.547, *P* < 0.001], postoperative serum maximum creatinine (Model 1: OR = 1.685, 95%CI = 1.469-1.932, *P* < 0.001; Model 2: OR = 1.635, 95%CI = 1.445-1.892, *P* < 0.001; Model 3: OR = 1.644, 95%CI = 1.473-1.836, *P* < 0.001), and postoperative serum minimum creatinine (Model 1: OR = 1.414, 95%CI = 1.082-1.844, *P* = 0.011; Model 2: OR = 1.441, 95%CI = 1.104-1.882, *P* = 0.007; Model 3: OR = 1.465, 95%CI = 1.174-1.828, *P* < 0.001) were all positively significantly associated with hospital mortality (Table 5). Moreover, serum creatinine before surgery was associated with hospital mortality (all *P* < 0.05, Table 5).

**Table 5:** Multivariable logistic regression analyses for hospital mortality associated with serum creatinine in cardiac surgery patients.

	**Model 1**		**Model 2**		**Model 3**	
**Variable**	**OR (95%CI)**	***P***	**OR (95%CI)**	***P***	**OR (95%CI)**	***P***
Postoperative serum initial creatinine	1.331 (1.112-1.593)	0.002	1.339 (1.116-1. 607)	0.002	1.361 (1.198-1.547)	<0.001
Postoperative serum maximum creatinine	1.685 (1.469-1.932)	<0.001	1.653 (1.445-1.892)	<0.001	1.644 (1.473-1.836)	<0.001
Postoperative serum minimum creatinine	1.414 (1.082-1.844)	0.011	1.441 (1.104-1.882)	0.007	1.465 (1.174-1.828)	0.001
Serum creatinine before surgery, mg/dl	1.198 (1.008-1.426)	0.040	1.199 (1.008-1.423)	0.040	1.212 (1.048-1.402)	0.010

*Model 1 was adjusted for age, height, SBP, DBP, RR, SpO2, temperature, congestive heart failure, cardiac arrhythmias, serum urea, renal failure, hypertension, liver disease*.

*Model 2 was adjusted for age, height, SBP, DBP, RR, SpO2, temperature, congestive heart failure, cardiac arrhythmias, serum urea, renal failure, hypertension, SOFA, valvular disease, liver disease*.

*Model 3 was adjusted for age, height, renal failure, hypertension, SOFA score*.

### Predictive Ability of Postoperative Serum Creatinine for Hospital Mortality

**Figure 1:**
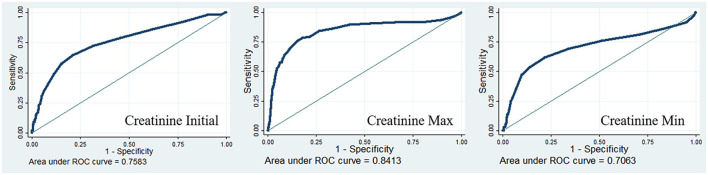
Receiver operating characteristic curves for postoperative serum creatinine in-hospital mortality of the cardiac patients.

The diagnostic value of postoperative serum creatinine was examined using ROC curves. The results showed that the diagnostic performance of creatinine was moderately good (initial creatinine AUC = 0.7583; maximum creatinine AUC = 0.8413; minimum creatinine AUC = 0.7063), especially the maximum creatinine (Figure 1).

### Comment

In the present study, the cardiac surgery patients in both the survivor and non-survivor groups had much higher creatinine values than the 0.3 mg/dl threshold. Moreover, positive correlations were observed between postoperative serum creatinine and lengths of ICU stay. In the fourth quartile of the initial postoperative creatinine value, hospital mortality significantly increased compared with that in the other quartiles. All the creatinine values, namely, creatinine initial, creatinine max, and creatinine min, were positively associated with hospital mortality and displayed moderately good diagnostic performance for hospital mortality.

Acute kidney injury, defined as a rapid decline in glomerular filtration rate that leads to elevated serum creatinine levels, imposes a heavy disease burden on cardiac surgery. Studies have shown that AKI after cardiac surgery is associated with longer hospital stays, higher costs, and higher mortality (14). The incidence of cardiac surgery-associated AKI (CSA-AKI) is between 20 and 40% (15, 16). Acute kidney injury complicates the recovery of heart surgery, impairs the brain, lung, and intestinal function, and increases the patient's risk of death during hospitalization by five times (17). The short-term mortality rate for CSA-AKI is between 15 and 30%, while the mortality rate for AKI-renal replacement therapy can be as high as 50-80% (18). In addition, those who recover from renal replacement therapy or mild AKI are more likely to develop chronic kidney disease in subsequent years than those who do not develop AKI (17).

Up to now, there are still many deficiencies in the understanding and diagnosis of AKI. The diagnosis of postoperative AKI depends on changes in serum creatinine levels from baseline or absolute reductions in urine volume, with the former parameter most often used by clinicians for practical reasons. In addition, the current research on creatinine as a poor prognostic factor after cardiac surgery is even more diverse. For the detection time node of creatinine, a previous study indicated that the serum creatinine value 48 h postoperatively is reflective of the outcome (19). A small decrease in serum creatinine showed a significant 30-day mortality increase in 8% of the patients (20). Moreover, studies have shown that even the smallest, ultra-short-term increase in serum creatinine (within 120 min after cardiac surgery) is associated with a large increase in 30-day mortality (11). These studies also support the conclusions of the present study. However, our study further explores the close relationship between 24 h postoperative creatinine value and short-term adverse outcomes after cardiac surgery. The role of the initial, maximum, and minimum values of creatinine was fully considered. Unlike this study, which focuses on short-term prognosis, studies by other researchers have shown that creatinine and cystatin C are also closely related to long-term adverse events (including death from any cause and dialysis) after cardiac surgery (21), and are powerful and independent of long-term mortality (22, 23). Our study also analyzed the role of preoperative serum creatinine in prognosis after cardiac surgery and found that it was also strongly associated with the length of ICU stay and was an independent risk factor for in-hospital death. Nevertheless, postoperative serum creatinine concentration was a risk factor for poor prognosis independent of preoperative serum creatinine. This suggests that cardiac surgery plays an important role in postoperative acute kidney injury in addition to the patient's renal basal state.

The pathophysiology of CSA-AKI is complex and multi-factorial. Ischemia-reperfusion injury, endogenous and exogenous toxins, metabolic factors, neurohormonal activation, inflammation, oxidative stress, and hemodynamics all play a role effect (14). Renal perfusion can be altered by many factors during surgery, and the cortical medullary junction and intramedullary tubules are often impaired (17). Other factors include sympathetic nervous system activation, the release of endogenous circulating catecholamines, and induction of the renin-angiotensin-aldosterone cascade, which may further impair renal oxygenation during surgery (17). Cardiac surgery can also cause inflammation in the kidneys and throughout the body. The mechanisms of increased inflammation during cardiac surgery are not fully understood, but contact activation of blood exposure to cardiopulmonary bypass (CPB), ischemia-reperfusion injury, and oxidative injury have all been implicated in inflammation. Cytokines and chemokines recruit concentrated granulocytes, macrophages, and lymphocytes into the renal parenchyma. The parenchymal infiltration and activation of these immune cells lead to the occurrence of AKI.

Data from animal studies suggest that the window for treatment of kidney injury is limited to the first few hours after injury, so it may be futile to treat it long after injury (24). Therefore, the early identification of kidney damage is critical for evaluating promising treatments. Multiple biomarkers of potential AKI have been investigated (e.g., neutrophil gelatinase-associated lipids calcitin, and cystatin C). However, to date, no study has been able to predict AKI with great accuracy. In addition, these biomarker tests are expensive and cannot be widely used in clinical practice. Recent evidence suggests that serum creatinine begins to rise shortly after surgery in cardiac surgery patients with AKI and may therefore be useful for early identification of patients with AKI (25). If the early postoperative elevation of serum creatinine can accurately identify patients with perioperative AKI, it would be ideal for early identification of high-risk patients for that the measurement of creatinine is quite easy and convenient. Currently, most AKI diagnosis uses KDIGO standards (26). The KDIGO standard defines AKI as an increase in serum creatinine by 0.3 mg/dl (≥26.5 mol/l) from baseline within the first 2 days after surgery, or a 50% increase in serum creatinine from baseline within the first 7 days after surgery, or a decrease in urine output of <0.5 ml/kg/h (lasts more than 6 h). Recent AKI diagnosis consensus criteria, including risk, injury, failure, loss, end stage renal disease (RIFLE), Acute Kidney Injury Network (AKIN), and KDIGO, all use changes in urine output and serum creatinine concentrations (26, 27). AKI diagnosis is most often considered by clinicians using serum creatinine measurements because it often occurs after cardiac surgery, and it is difficult to accurately record hourly urine output. The creatinine endpoint is better validated than the oliguria endpoint. However, it takes hours or even days to diagnose AKI after renal injury, and may be insensitive to mild renal injury because the kidney can maintain function by maintaining glomerular filtration when the nephron is damaged. However, small changes in serum creatinine may reflect non-AKI-specific sustained systemic inflammatory process. Nevertheless, the 0.3 mg/dl threshold is closely related to poor results (20, 28).

The accurate prediction of AKI provides opportunities for clinicians to optimize high-risk patients, increase monitoring, include patients in clinical trials, and carry out preventive and therapeutic treatments. Serum creatinine is a very low-cost marker that can achieve very early risk stratification. This study suggests that creatinine at 24 h after surgery seems to be a key biomarker for predicting the mortality and clinical outcome of patients after cardiac surgery. A reliable and sensitive outcome indicator is essential for judging the mortality rate and fully understanding the condition of the patient. In addition, the prediction of AKI helps to introduce preventive measures more quickly.

Research believes that by improving the awareness of early prevention and better management, the burden of AKI can be reduced and the quality of care can be improved. Strategies to prevent AKI are part of the daily management of cardiac surgery patients. For example, intravenous fluid management, surgical and extracorporeal circulation techniques, and hemodynamic stability will all affect the development of AKI (17). In clinical trials, pharmacological and many non-pharmacological treatments have largely failed to reduce AKI associated with cardiac surgery, although some treatments may be effective for specific patients. In addition, a lot of the risk factors for AKI are unchangeable, such as hyperlipidemia, hypertension, age, peripheral vascular disease (29), and so on. But some factors are unique to anesthesia, surgery, and ICU management, so doctors should be aware of these controllable factors and take steps to reduce their harmful effects. Compared with non-cardiac surgery, the uniqueness of cardiac surgery contains extracorporeal circulation (CPB), aortic clipping, high infusion rate, high-dose use of exogenous vasopressors, and infusion of exogenous blood products, can significantly increase the AKI risk. These factors can change renal perfusion, result in ischemia and reperfusion cycles, lead to oxidative injury, and exacerbate renal and systemic inflammation, all of which are related to the development of AKI (30). Currently, the reduction of AKI after cardiac surgery and its impact on patient morbidity is limited to hemodynamic procedures with close attention to venous resuscitation strategies, including target-directed therapy and balanced saline solution administration, reducing extracorporeal circulation exposure, and identifying and mitigating modifiable risk factors (17).

Although the study has reported on the relationship between postoperative creatinine and the prognosis of cardiac surgery, the large sample size, and 4-year follow-up data of this center can provide significant data for in-depth exploration of the relationship between creatinine and the prognosis of cardiac surgery. However, this study has some limitations. First, this was a single-center retrospective non-randomized study. Second, the definition of surgical types and comorbidities based on the ICD-9-CM code in this study may not be specific enough. And some important information was lacking in the database, such as continuous ven-venous hemofiltration, preoperative risk score, intraoperative details, etc. These were the limitations of the database itself. Thirdly, the patients included in the database were from 2001 to 2012, of which the time interval was relatively long. Due to the privacy protection of the database, the patient admission time provided by the database was hidden, and only the relative time such as the length of hospitalization, the length of the ICU could be obtained, and the accurate time of admission and operation could not be obtained. Therefore, it was difficult to distinguish the differences in the characteristics, indications, and prognosis of patients with the same condition in such a long time interval.

In summary, we showed a positive relationship between the initial, maximum, and minimum values of postoperative creatinine during the lengths of ICU stay and prognosis. Our study demonstrated a novel proposal of using postcardiac serum creatinine levels (including initial, maximum, and minimum) as outcome indicators.

## Data Availability Statement

The raw data supporting the conclusions of this article will be made available by the authors, without undue reservation.

## Ethics Statement

The studies involving human participants were reviewed and approved by the Institutional Review Boards of the Massachusetts Institute of Technology (Cambridge, MA, USA). Written informed consent for participation was not required for this study in accordance with the national legislation and the institutional requirements.

## Author Contributions

JH, LS, and SH: conceptualization. JH, SH, LS, and JY: data curation. JH, LS, YA, and ZW: statistical analysis. YA, JY, and ZW: methodology. ZW and JH: funding acquisition. All authors contributed to the article and approved the submitted version.

## Funding

This study was funded by the National Natural Science Foundation of China (81900294, 81770319, 81570039, and 82070297).

## Conflict of Interest

The authors declare that the research was conducted in the absence of any commercial or financial relationships that could be construed as a potential conflict of interest.

## Publisher's Note

All claims expressed in this article are solely those of the authors and do not necessarily represent those of their affiliated organizations, or those of the publisher, the editors and the reviewers. Any product that may be evaluated in this article, or claim that may be made by its manufacturer, is not guaranteed or endorsed by the publisher.
