# Supplementary material for: Postoperative Serum Creatinine Serves as a Prognostic Predictor of Cardiac Surgery Patients
Source: Front Cardiovasc Med. 2022 Feb 16;9:740425. doi: 10.3389/fcvm.2022.740425 (PMC8888823; doi:10.3389/fcvm.2022.740425)
Supplement: Supplementary file 1 [file Table_1.docx]

| **Supplement Table 1 The correlation of serum creatinine before surgery with** postoperative serum creatinine | | |
| --- | --- | --- |
|  | Rho | *P* value |
| Postoperative serum initial creatinine, mg/dL | 0.752 | <0.001 |
| Postoperative serum maximum creatinine, mg/dL | 0.712 | <0.001 |
| Postoperative serum minimum creatinine, mg/dL | 0.768 | <0.001 |
